# Primary care follow up of patients discharged from the emergency department: a retrospective study

**DOI:** 10.1186/1471-2296-5-16

**Published:** 2004-08-07

**Authors:** Shlomo Vinker, Eliezer Kitai, Yaacov Or, Sasson Nakar

**Affiliations:** 1 The Department of Family Medicine, Clalit Health Services, Central District, Rishon Lezion, Israel; 2 The Family Medicine Department, Sackler Faculty of Medicine, Tel Aviv University, Tel Aviv, Israel; 3 Emergency Department, Kaplan Medical Center Rehovot, Affiliated with the Hebrew University and Haddasa Faculty of Medicine, Rehovot, Israel

## Abstract

**Background:**

The visit to the emergency department (ED) constitutes a brief, yet an important point in the continuum of medical care. The aim of our study was to evaluate the continuity of care of adult ED visitors.

**Methods:**

We retrospectively reviewed all ED discharge summaries for over a month 's period. The ED chart, referral letter and the patient's primary care file were reviewed. Data collected included: age, gender, date and hour of ED visit, documentation of ED referral and ED discharge letter in the primary care file.

**Results:**

359 visits were eligible for the study. 192 (53.5%) of the patients were women, average age 54.1 ± 18.7 years (mean ± SD). 214 (59.6%) of the visits were during working hours of primary care clinics ("working hours"), while the rest were "out of hours" visits. Only 196 (54.6%) of patients had a referral letter, usually from their family physician. A third (71/214) of "working hours" visits were self referrals, the rate rose to 63.5% (92/145) of "out of hours" visits (p < 0.0001). The ED discharge letter was found in 50% (179/359) of the primary care files. A follow-up visit was documented in only 31% (111/359). Neither follow up visit nor discharge letter were found in 43% of the files (153/359).

**Conclusions:**

We have found a high rate of ED self referrals throughout the day together with low documentation rates of ED visits in the primary care charts. Our findings point to a poor continuity of care of ED attendees.

## Background

The emergency department (ED) is intended to treat medical urgencies or emergencies, but a large proportion of visits are due to problems that could be treated in the primary care setting [1,2]. ED services are available 24 hours a day while primary care facilities have limited service hours. In the Israeli health system patients can be referred to the ED by their family practitioner, or by other community health providers, or be self referred. Recently many out of hours community based services have been established but without a significant reduction in visiting rates to in-hospital ED.

The visit to the ED constitutes a brief, yet an important point in the continuum of medical care. In today's era of cost effectiveness and increasingly competent primary care physicians, ambulatory investigation, treatment and follow-up have largely replaced prolonged and costly hospitalizations [3,4]. The ED visit however, remains a cross-road which may mark a sudden change in the patient's medical condition. In many cases it may result in introducing new medications, withdrawing others and recommendation of further investigations. The family practitioner is the one expected to coordinate and carry out the treatment and follow-up. The new information given from the ED should be effectively delivered to the family practitioner, the modality usually used is the discharge letter.

The continuation of treatment between hospital departments and the primary care physician had been issued in several studies using discharge letters audit [5-7]. Raval et. al. assessed the adequacy of the discharge summary in reporting important investigative results and future management plans in patients hospitalized and discharged with a diagnosis of heart failure [5]. They found substantial inadequacies in communicating to the community physician that may have implications for continuity of care and subsequent clinical outcome. Wilson et. al. examined the reliability, effectiveness, accuracy and timeliness of hospital to general practitioner information transfer by discharge summaries. In a retrospective audit of 569 patient discharge summaries and related medical records they found that summaries written for patients discharged from hospital were estimated to be received by the patient-nominated general practitioner in 27.1% of cases [6]. Bolton et al assessed the quality of communications between hospitals and general practitioners. The general practitioner's(GP) name was recorded in 88% of audited records. Few inaccuracies were detected in the medications recorded in the discharge summaries, and on contrary to Wilson et al 77% of discharge summaries were received by the GP [7]. The continuity of care between the ED and the primary care physician had been assessed for children with asthma [8] but we did not find data about the continuity of care for adults.

To evaluate the continuity of care after ED visits, we evaluated the ED referral and discharge letters, their content, and the documentation of the ED visit in the patients' primary care files. We have focused on discharges from the internal medicine ED. We expected that in these cases the patients would be followed up by their family practitioner.

## Methods

The study was conducted in the district medical center (Kaplan), serving more than 500,000 inhabitants, and in 12 primary care clinics (32 family practitioners), of The Clalit Health Services in the Rehovot region, Israel.

In Israel the entire population have a national health insurance by law and each citizen can choose to be a member of one of four HMOs. Every member of the Clalit Health Services, the largest HMO in Israel, is registered to a single family physician, and have a medical record in his physician's clinic. Visits to the emergency department are regulated in the national health insurance law. A referral by a physician or by ambulance is free of charge, but this referral should be with a referral letter and not by a phone call to the ED or to the patient. A self referral may cost the patient a co-payment of up to 100 USD.

We reviewed retrospectively all the charts of the ED visits for a period of one month, excluding the visits to the pediatric and the gynecologic-obstetrics EDs (see flow-chart 1). 5,898 visits documented that month, resulted in 4,256 discharges and 1,642 hospitalizations. There were 1,564 discharges from the general ED. Trauma, surgery and orthopedics accounted 2,209 discharges and the rest 483 were from other specialties (ophthalmology, ENT, dermatology etc.).

Inclusion criteria were: visit to the general ED, age above 18 years, discharge to the community (not hospitalized) at that visit, living and getting medical care in a family medicine group practice in the Rehovot region. Visits due to accidents, trauma, surgery, orthopedics, ENT, ophthalmology and other specialities were excluded from the study. The 1,564 discharges from the general ED were reviewed and 359 were found to be eligible to this study.

Two physicians reviewed independently each ED medical chart. Data extracted included: age and gender of the patient, attendance date and hour, self referral, or a referral by a physician and the final diagnosis in the discharge letter. In cases of referrals the content and format of the referral letter were assessed, including: hand writing quality and whether the referring physician referred the patient with a specific question (for example: rule out new onset angina pectoris, suspected pneumonia, please make a chest X ray etc.).

Continuity of communication and care: The primary care files of ED visitors were retrieved and checked for the existence of the ED discharge letter and comments about the visit in the follow-up chart. If the discharge letter and / or any comment on the ED visit in the follow-up chart had been found the case was defined as "a case with good continuity of care".

The cases in which the family physician was the referring physician we looked for documentation of the encounter prior to ED attendance.

Visits to the ED were divided into "working hours" visits – when the visit took place during working hours of primary care clinics in the community (Sunday to Thursday from 08:00 to 20:00, and Friday 08:00 to 14:00), and "out of hours" visits when primary care clinics in the community are closed (from 20:00 to 08:00 weekdays, and weekends from 14:00 on Friday to 08:00 on the following Sunday).

A referral letter was defined as "any document written by a medical authority in the community prior to the index ED visit", including referrals from family practitioners, other practitioners in the community and arrival by an ambulance.

A recurrent visit to the ED was defined as a patient's visit to ED within less than two weeks from a previous visit with the same complaint, when in both cases the patient was not hospitalized.

Diagnoses at discharge were coded for a specific diagnosis and for the system involved according to the ICPC coding system.

Statistical analysis: Data was analyzed using distribution analysis and χ^2 ^tests to investigate the association between categorized variables. Student's t tests were used to analyze continuous variables. The analysis was performed using the SPSS package.

## Results

During the study period there were 359 ED visits that were eligible to be included in the study (table 1, flow chart 1). . 214 (59.6%) visited the ED during the "working hours" of primary care clinics, 28 (7.8% of all visits) were recurrent visits to the ED.

**Table 1:** Data on 359 visitis to the Emergency Department

	All visits (359)	Referral letter (196)	Self referral (163)	P Value*
Gender				
Women -	192 (53.5%)	106 (54%)	86 (52.7%)	NS
Men –	167 (46.5%)	96 (46%)	77 (47.3%)	
Age (years, mean ± SD)	54.1 ± 18.7	55.1 ± 19.0	52.9 ± 18.4	NS
Age distribution				
<45	127 (35.4%)	68 (34.7%)	59 (36.2%)	NS
46–65	105 (29.3%)	53 (27%)	52 (31.9%)	
66–75	63 (17.5%)	38 (19.4%)	25 (15.3%)	
>75	64 (17.8%)	37 (18.9%)	27 (16.6%)	
Visit time				
Working hours –	214 (59.6%)	143 (73%)	71 (43.6%)	<0.0001
Out of hours –	145 (40.4%)	53 (27%)	92 (56.4%)	

* – p Value for comparison between patients with referral letter and self referrals

Out of all ED visits only 196 (54.6%) patients had a referral letter, the rest were self-referrals. Referral letters were mainly from the family practitioner (147/196, 75%), 14 (7%) from other practitioners in the community, and 35 (18%) of referrals were by ambulance. The referral letters from the community were legible in 43.4% (70/161), 46.5% (75/161) were barely legible and 10% (16/161) illegible. In only 25/161 letters (15.7% of the referrals) a specific question was asked by the referring physician and in another 32 (20.2%) there was only a general question.

The main diagnostic groups according to the ICPC were: respiratory (15.7%), digestive system (18.1%), musculo-skeletal (15.2%) and cardio-vascular (11%). In 9.6% of the cases the discharge letter did not contain a specific diagnosis and the diagnosis fell in the "general" category. The most common specific diagnoses were: chest pain (5.9%), abdominal pain (3.9%), other respiratory tract infections (3.7%), asthma (3.1%), back pain (2.8%), COPD exacerbation (2.8%) headache (2.5%), nephrolithiasis (2.5%), vertigo or dizziness (2.5%) and gastroenteritis (2.3%).

The "out of hours" visitors tended to be younger (52.2 ± 17.5 vs. 55.4 ± 19.5, p = NS) (table 1). A third of the "working hours" visits (71/214) were self referrals as opposed to 63.5% (92/145) of "out of hours" visits (p < 0.0001). Table 2 compares the referring practitioners according to ED visiting hours.

**Table 2:** Comparison between the source of referral, in 196 visits according to ED visit hours*

The referring practitioner	"Working hours"**	"Out of hours"	Total
The Family practitioner	125 (87.5%)	22 (41.5%)	147
By ambulance	16 (11%)	19 (36%)	35
Other practitioner***	2 (1.5%)	12 (22.5%)	14
Total	143 (100%)	53 (100%)	196

* – p < 0.0001 ** – "working hours" visits – when the visit was during the working hours of primary care clinics in the community *** – private practitioners, and out of hours commmunity emergency clinics

In 147 cases the reffering physician was the family physician, documentation of the ED referral was found in 32% (47/147) of primary care files. The ED discharge letter was found in 50% (179/359) of the primary care files. A follow-up visit was documented in only 31% (111/359). Neither follow up visits nor discharge letters were found in 43% of the files (153/359). No associations between clinic characteristics (size, place) or family practitioner qualification and ED visit documentation was found.

## Discussion

The Emergency Department (ED) acts as a link between community and hospital based medicine. In Israel a patient who needs non elective admission to a hospital unit must pass through the ED, either with a referral note from a medical practitioner, or as a self referral. Most ED visits, however, do not result in hospitalization, and many could be regarded as primary health care problems [1,2,9]. These patients are discharged directly from the ED to the community and further care of the family practitioner. A visit to the ED is generally not prompted by a benign complaint; The most common reasons for referral include, chest pain, asthma exacerbations and nephrolithiasis, subsequent follow up by the family practitioner can be vital. It was found that most children do not have outpatient follow-up after an ED asthma visit [8]. However, those patients that present for outpatient follow-up have an increased likelihood for repeat ED asthma visits, and this visit should be a key opportunity to prevent future ED asthma visits.

The increasing role played by the ED in treating primary care problems has been discussed in a number of recent articles [9-11].

One aspect, which is important to the ED team, is the logistics and manpower needed to optimize the treatment of these non-urgent patients in ways that will not interfere with emergencies yet providing them adequate care. It is unclear whether the capability and quality of primary care services in the ED should be improved and compete with the community family physicians. This is true especially in Israel where there is a universal national health insurance and every patient can have a personal family practitioner.

The continuity of comprehensive management is expected from the family practitioner, and is gaining importance nowadays [12]. To achieve this goal the communication between health care providers who treat the patient is mandatory. In the case of the ED visit, where we found many self referrals and referrals from other physicians, it becomes even more important.

The modes of communication are the referral letter and the discharge letter. We have found that the referral letter can be improved both in style (printed instead of illegible hand writing) and content (the referring physician should define and clarify the reasons for referral and his expectations). These problems exist in discharge letters as well [13].

Documentation in the primary care file was poor, only one third of referrals were documented and less than 60% of discharges. This figure is between the 27%–77% that was found by others [6,7]. A possible bias is that some follow-up visits were to specialists. But in the case of discharge from the general medicine ED we presume that most patients were advised to return to their family physicians.

It is well known that medical notes are poor in other areas, Miller et al [14] found documentation of only 15% of prescriptions given by family practitioners. They explained one of the causes for this discrepancy as the need of double writing (both the prescription itself and in the medical notes). By introducing carbon copy prescriptions, they achieved an 82% documentation rates in patients' files. Opila [15] found documentation in out patient medical records greatly improved after employing quality control and a feedback system.

With the introduction of computerized medical files in primary care clinics in our region, the need of "double writing" will disappear, and this in turn should dramatically improve documentation rate of referrals and discharges to the ED; particularly if a computerized reminder system is used to encourage follow up of referrals by the family practitioner.

### Limitations

Israeli health care system works in regard to ED use quite different from the US and other countries. Likewhise these results may not automatically be generalized to other health care systems. This study described the written communication between the emergency department and the primary care physician, which is the first and mandatory step in establishing continuation of care. This is only one of the four dimensions of continuity of care in family practice: chronological, geographical, interdisciplinary, and interpersonal [16]. Each of these dimensions may influence the quality of care and be evaluated and studied. Further study is needed to prove the link between documantion of ED visit and good contuniuity of care. Large scale prospective intervention studies are needed to prove that continuity of care between ED and the primary care physician improves outcome and saves money.

## Conclusion

ED visits may have important implications for the patient and his family practitioner. The high rate of ED self referrals together with low documentation rates of ED visits in the primary care charts result in poor continuity of care of ED visitors.

## Competing interests

None declared.

## Authors' contributions

All authors read and approved the final manuscript.

VS Conceived and designed the study, participated in the collection, analysis and interpretation of data and drafted the manuscript. KE Participated in the statistical analysis, interpretation of data and draft of the manuscript.OY participated in the design of the study, data collection and interpretetio hand draft of the manuscript. SN participated in the design of the study, interpretation of data and draft of the manuscript. All authors have read and approved the final manuscript.

**Figure 1:**
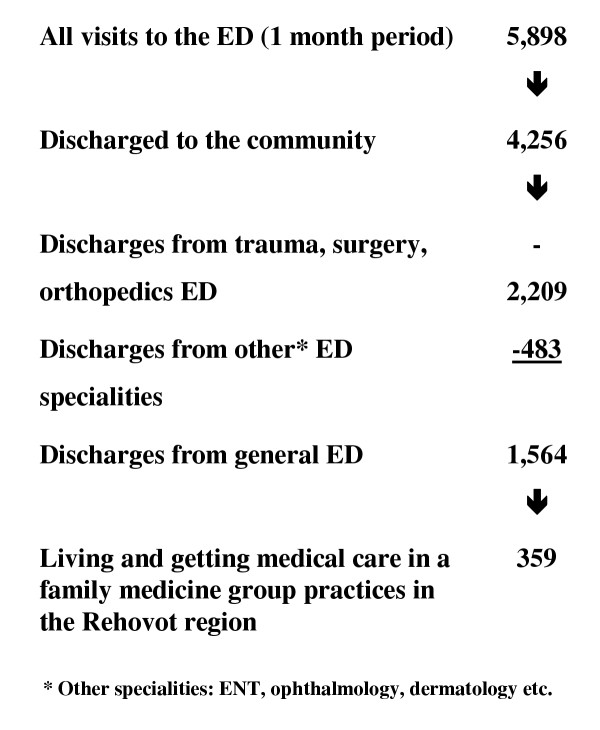
Emergency department (ED) visits that included in the study

## Pre-publication history

The pre-publication history for this paper can be accessed here:


